# Supplementary material for: Research on aging behaviors of insulating silicone oil for cable terminals based on chromatographic and spectroscopic analysis
Source: PLoS One. 2025 Nov 7;20(11):e0334552. doi: 10.1371/journal.pone.0334552 (PMC12594329; doi:10.1371/journal.pone.0334552)
Supplement: Attachment — Submitted filename: Comments 2025-04-07.docx [file pone.0334552.s002.docx]

**Comments**

Research studies on aging behaviours of insulating silicon oil for cable terminals based on chromatographic analysis are essential to establish operational quality control measures. In this study, the authors have employed Gas Chromatography (GC) and Fourier Transform Infrared (FTIR) Spectroscopy to examine the aging characteristics of insulating silicon oil and established some criteria for fault diagnosis of insulating silicon oil. The study is meaningful; however, some issues need to be addressed before its publication:

1. The write-up of this manuscript doesn’t sound scientifically, the authors should follow proper scientific writing methods.
2. The title of the manuscript is not clear, consider revising it. The authors may consider a title such as “Research study on aging behaviours of insulating silicon oil for cable terminals based on chromatographic analysis” or improve it better.
3. The manuscript has some grammar and typo mistakes that need to be improved or corrected. For example, 140℃, 350ppm, etc. There should be a space between a number and a unit like 140 ℃ and 350 ppm. Also, in some areas there is no space after a period or full stop like “discharges.Considering” (line 132), and “interference.Place” (line 134). Make the necessary corrections throughout your manuscript.
4. The manuscript lacks consistency, for example, “Fig. 1.” and “Fig 2.” are used interchangeably. The authors should observe consistency throughout the manuscript.
5. In section 2 “Experimental and simulation methods”, which simulation method was used in this work and for what purpose? The ball and stick structure of silicon in Figure 1 is not clear, redraw and make it colourful.
6. The basic parameters given in Table 1 were they determined by the authors or other researchers? If authors, explain how they are determined or derived, and if other researchers, a reference is required.
7. Why the aging characteristics of the insulating silicon oil are tested only at 140 ℃ instead of a range of temperatures?
8. Figures 2 to 7, Tables 2 to 5, and Equations 1 to 3 are not mentioned or cited anywhere in the text. Figures, Tables, and Equations should be part of the text.
9. Why it is worthy reporting Figures 3 and 4 in the manuscript?
10. What is the unit for the y-axis in Figure 5? The y-axis title text direction should be changed.
11. Figures resolution is not good, consider redrawing them and make attractive.
12. Equations 1 to 3 are confusing; they should be properly written.
13. Generally, the manuscript in its current version lacks sufficient novelty. The author(s) should revise or reformulate the abstract, introduction, methodology, and conclusion to clearly show the strength and novelty of the manuscript.
