# Supplementary material for: Research on aging behaviors of insulating silicone oil for cable terminals based on chromatographic and spectroscopic analysis
Source: PLoS One. 2025 Nov 7;20(11):e0334552. doi: 10.1371/journal.pone.0334552 (PMC12594329; doi:10.1371/journal.pone.0334552)
Supplement: Attachment — Submitted filename: Response to Reviewers.docx [file pone.0334552.s004.docx]

**Revision Report for Manuscript** **PONE-D-25-13265**

**Dear Editors:**

We gratefully appreciate both editors and reviewers for their careful reading, helpful comments, and constructive suggestions, which have significantly improved the quality of our manuscript entitled “**Research on Aging and Oil Chromatographic Characteristics of Insulating Silicone Oil for Cable Terminals**” (**Manuscript Number: PONE-D-25-13265**).

We have carefully considered all comments from the reviewers and revised our manuscript accordingly. In the following section, we summarize our responses to each comment from the reviewers. We believe that our responses have well addressed all concerns from the reviewers. Here, we would like to re-submit the revised manuscript to *PLOS ONE*. Hope these revisions could present a better view to the readers and make the paper acceptable for publication in the journal.

Looking forward to hearing from you soon. Have a nice day.

Best regards,

The Authors

Electric Power Research Institute of State Grid Jiangsu Electric Power Co,

Nanjing, 211103, China

+86-17805166516

zhangweijsdl@163.com

**Response to Reviewer #1:**

**General comments:**

Research studies on aging behaviors of insulating silicon oil for cable terminals based on chromatographic analysis are essential to establish operational quality control measures. In this study, the authors have employed Gas Chromatography (GC) and Fourier Transform Infrared (FTIR) Spectroscopy to examine the aging characteristics of insulating silicon oil and established some criteria for fault diagnosis of insulating silicon oil. The study is meaningful; however, some issues need to be addressed before its publication:

**Answer:**

Thank you very much for your positive feedback and kind words regarding our revisions. We are grateful for your continued support and thoughtful comments, which have significantly contributed to strengthening the manuscript. We will carefully address all the comments and make the necessary adjustments to ensure the paper meets the required standards. We appreciate your time and effort in reviewing the manuscript and look forward to submitting the final version with all corrections made.

We have modified the manuscript with a clearer expression and show the changes made in the revised paper in red color. The specific comments were answered point by point as follows.

**Specific comments 1:**

The write-up of this manuscript doesn’t sound scientifically, the authors should follow proper scientific writing methods.

**Answer:**

Thank you for your valuable feedback. We have carefully revised the entire manuscript to improve its scientific rigor and clarity. Colloquial or informal expressions have been replaced with academically appropriate phrasing, and the logical flow of the arguments has been significantly enhanced. Moreover, we have ensured consistent use of terminology, corrected grammatical issues, and employed passive voice and formal structures in line with scientific writing standards. We believe these revisions greatly improve the readability and professionalism of the manuscript. We hope these revisions will adequately address your comments.

**Specific comments 2:**

The title of the manuscript is not clear, consider revising it. The authors may consider a title such as “Research study on aging behaviours of insulating silicon oil for cable terminals based on chromatographic analysis” or improve it better.

**Answer:**

Thank you for your suggestion. We agree that the original title was not clear and we have revised it to:

**“Research on Aging Behaviors of Insulating Silicone Oil for Cable Terminals Based on Chromatographic and Spectroscopic Analysis”**

This new title better reflects the core aspects of the study, we hope this title will improve the clarity and precision of the manuscript.

**Specific comments 3:**

The manuscript has some grammar and typo mistakes that need to be improved or corrected. For example, 140℃, 350ppm, etc. There should be a space between a number and a unit like 140 ℃ and 350 ppm. Also, in some areas there is no space after a period or full stop like “discharges.Considering” (line 132), and “interference.Place” (line 134). Make the necessary corrections throughout your manuscript.

**Answer:**

We sincerely thank the reviewer for pointing out the grammar and typo issues in our manuscript. In response, we have carefully reviewed the entire manuscript and made the necessary corrections, including insert spaces between numbers and units and add spaces after periods and commas, et al. We sincerely apologize for the oversight and appreciate your attention to detail, which has helped improve the quality of our submission.

**Revision:**

**Abstract**

*Line19-22*

*Accelerated thermal aging experiments (140 °C, 30 days) were conducted to simulate long-term aging of silicone oil. By integrating partial discharge, high-energy discharge, and breakdown experiments, the gas generation patterns of silicone oil under different stresses were systematically analyzed.*

*Line141-159*

*Oil chromatography analysis primarily detects seven gas components generated during aging degradation: H₂: Reflects moisture or hydrocarbon decomposition in insulation materials. CH₄, C₂H₆, C₂H₄, C₂H₂: Characterize hydrocarbon pyrolysis or corona discharge. CO, CO₂: Indicate decomposition of oxygen-containing groups in silicone oil. C₂H₂: A signature gas for high-energy discharges. Considering the instrument’s single-measurement requirement of 40 mL, extract 60 mL of post-discharge silicone oil sample. Then inject 1 mL of high-purity nitrogen as a carrier gas to assist gas separation and avoid oxygen interference. Place the sample in a constant-temperature shaker at 50 °C for 20 minutes to promote gas liberation. After shaking, use a gas-tight syringe to extract gas from the sample headspace, avoiding external air contamination, and immediately proceed to measurement. A chromatographic column selectively adsorbs gases based on polarity. Low-molecular-weight gases (H₂, CH₄) with weak adsorption elute first. Polar gases (CO, CO₂) interact strongly with the stationary phase, prolonging retention time. Hydrocarbon gases (C₂H₆, C₂H₄, C₂H₂) are separated sequentially by carbon chain length and double bond count. The thermal conductivity detector is used for detecting flameless combustion gases such as H₂, CO, and CO₂. Quantifies hydrocarbons (CH₄, C₂H₆) by ionizing them in a hydrogen flame and measuring the resulting current. Gas species are identified by comparing peak retention times with standard gas calibrations. Concentrations are calculated using the internal standard method, correlating peak areas with calibration curves.*

*Line213*

| **Time (days)** | **H₂ Concentration (ppm)** | $\ln\left( \frac{C_{max}}{C_{max}-C} \right)$ |
| --- | --- | --- |
| 5 | 80.77 | 0.12 |
| 10 | 150.2 | 0.32 |
| 30 | 323.3 | 0.87 |
| Assuming $C_{max}$ = 350ppm, The fitting result is *k* ≈ 0.029 day ⁻ ¹. | | |

**Specific comments 4:**

The manuscript lacks consistency, for example, “Fig. 1.” and “Fig 2.” are used interchangeably. The authors should observe consistency throughout the manuscript.

**Answer:**

Thank you for highlighting this inconsistency. We have carefully revised the manuscript to ensure consistency in formatting throughout. All figure and table references have been updated to the standardized format “Fig X.” and “Table X.”. We ensured these changes are applied uniformly across the text to align with academic writing conventions. We sincerely apologize for the oversight and appreciate your attention to detail, which has helped improve the quality of our submission.

**Specific comments 5:**

In section 2 “Experimental and simulation methods”, which simulation method was used in this work and for what purpose? The ball and stick structure of silicon in Figure 1 is not clear, redraw and make it colourful.

**Answer:**

Thank you for your valuable comment. We apologize for the confusion caused by the section title. In fact, this work focuses solely on experimental investigations, and no simulation methods were employed. To avoid misunderstanding, we have revised the section title to “Experimental Methods” accordingly. Regarding Figure 1, we appreciate the reviewer’s suggestion. The original image has been redrawn using improved resolution and a clearer color scheme to enhance its visual clarity and readability. The updated figure is included in the revised manuscript.

**Revision:**

**Specific comments 6:**

The basic parameters given in Table 1 were they determined by the authors or other researchers? If authors, explain how they are determined or derived, and if other researchers, a reference is required.

**Answer:**

Thank you for this important clarification request. The parameters listed in Table 1 (e.g., density, kinematic viscosity, flash point, etc.) are not obtained experimentally by the authors but are sourced from the technical datasheet provided by the manufacturer, Wacker Chemie AG. We have updated the manuscript accordingly to state this explicitly.

**Revision:**

*Line80-85*

*The silicone oil sample selected for testing was AK-50 insulating silicone oil produced by Wacker Chemie AG, Germany. Its primary component is polydimethylsiloxane (PDMS), featuring a linear chain structure composed of alternating silicon and oxygen atoms, with each silicon atom bonded to two methyl groups. The basic molecular structure is illustrated in Fig 1. The key physical and chemical properties of the silicone oil sample are summarized in Table 1(The technical datasheet provided by the manufacturer, Wacker Chemie AG).*

**Specific comments 7:**

Why the aging characteristics of the insulating silicon oil are tested only at 140 ℃ instead of a range of temperatures?

**Answer:**

Thank you for your important comments. In this study, 140 ℃ was deliberately chosen as the aging temperature to represent an accelerated thermal aging condition, approximately 3 times higher than the typical operating temperature range (40-50 ℃) for insulating silicone oil in practical high voltage cable terminal. This approach allows for the efficient observation of thermal degradation and moisture-related changes within a manageable experimental timeframe. The suggestions from the reviewers are very correct. In our subsequent experimental plan, we will consider adding multiple heat aging temperature points for the experiments to study the effects of different heat aging temperatures on the insulating oil.

**Specific comments 8:**

Figures 2 to 7, Tables 2 to 5, and Equations 1 to 3 are not mentioned or cited anywhere in the text. Figures, Tables, and Equations should be part of the text.

**Answer:**

Thank you for carefully pointing out this important oversight. We have thoroughly revised the manuscript to ensure that all figures (Figs 2 to 7), tables (Tables 2 to 5), and equations (Equations 1 to 3) are clearly cited and discussed at appropriate points in the manuscript. These elements have now been fully integrated into the narrative to support and clarify the experimental results and analysis. We sincerely apologize for the omission and appreciate the reviewer’s detailed attention, which has helped us improve the manuscript's structure and readability.

**Revision:**

*Line112-114*

*The silicone oil is sealed in a clean bottle and placed in a drying oven for high-temperature thermal aging. Fig 2 shows actual photographs of the oven and the silicone oil under different aging states.*

*Line126-127*

*The schematic diagram of the experimental circuit and the physical photograph of the needle-plate electrode are shown in Fig 3.*

*Line150-151*

*The schematic flow diagram illustrating the principle of oil chromatography detection is shown in Fig 4.*

*Line188-189*

*The oil chromatography data detected during continuous 30-day thermal aging are presented in Fig 5.*

*Line261-262*

*The infrared spectrum of the new silicone oil is presented in Fig 6, obtained from infrared spectroscopy analysis.*

*Line273-274*

*The results shown in Fig 7 indicate that no significant chemical bond breakage occurred in the silicone oil after 30 days of aging.*

*Line212*

*Taking H₂ as an example, The example calculation for H₂ is presented in Table 2:*

*Line224-225*

*The bond energies of other gases calculated using the aforementioned equations are presented in Table 3:*

*Line234-235*

*The oil chromatography data for different discharge stages are presented in Table 4.*

*Line249-250*

*The oil chromatography data after breakdown are presented in Table 5.*

*Line268-269*

*As can be seen from the figure above, the main absorption peaks of the silicone oil are listed in Table 6, along with the analysis of the corresponding functional groups.*

*Line206-207*

*Equation 1 and 2 are the first-order kinetic reaction formula, and equation 3 is Arrhenius formula.*

**Specific comments 9:**

Why it is worthy reporting Figures 3 and 4 in the manuscript?

**Answer:**

Thank you for your question. Figures 3 and 4 serve important roles in illustrating the experimental setup. Figure 3 provides both a schematic diagram of the partial discharge (PD) test circuit and a photo of the needle-plate electrode, which are critical for understanding how discharge conditions were established and controlled. Figure 4 illustrates the configuration of the gas chromatography testing system, helping readers understand the sampling, degassing, and gas analysis procedures used for identifying degradation gases in silicone oil. These figures enhance the methodological clarity and reproducibility of our work and are therefore essential to retain in the manuscript.

**Specific comments 10:**

What is the unit for the y-axis in Figure 5? The y-axis title text direction should be changed.

**Answer:**

Thank you for bringing up this matter. We have revised Figure 5 accordingly and added unit annotations.

We hope this revision satisfies your concerns.

**Revision:**

*Line183*

*Line185*

**Specific comments 11:**

Figures resolution is not good, consider redrawing them and make attractive.

**Answer:**

Thank you for pointing this out. We have reviewed all figures and are in the process of redrawing them with enhanced clarity and consistency. All revised figures are exported at high resolution with improved visual contrast, uniform font styling, and clear annotations. These updates ensure professional presentation quality and facilitate better interpretation by readers.

We hope this revision satisfies your concerns.

**Revision:**

*Line183*

*Line185*

*Line266*

*Line271*

**Specific comments 12:**

Equations 1 to 3 are confusing; they should be properly written.

**Answer:**

Thank you for this valuable comment. We have revised Equations (1) to (3) to adopt a clear and consistent scientific format. Each equation is now properly centered, numbered.

We hope this revision satisfies your concerns.

**Revision:**

$$\frac{dC}{dt}=k\times\left( C_{max}-C \right) (1)$$

$$\ln\left( \frac{C_{max}}{C_{max}-C} \right)=kt (2)$$

$$k=A\times e^{-\frac{E_{a}}{RT}} (3)$$

**Specific comments 13:**

Generally, the manuscript in its current version lacks sufficient novelty. The author(s) should revise or reformulate the abstract, introduction, methodology, and conclusion to clearly show the strength and novelty of the manuscript.

**Answer:**

We thank the reviewer for this important suggestion. In response, we have substantially revised the abstract, introduction, methodology, and conclusion to highlight the scientific contributions and novelty of our work. The major novelties are now clearly emphasized as follows:

This is the first study to propose gas ratio-based fault diagnosis criteria specifically for insulating silicone oil, which are not covered by existing standards developed for transformer oil. We introduce quantitative gas ratios (e.g., H₂/CH₄, C₂H₄/C₂H₆, C₂H₂/C₂H₄) as effective indicators of fault types in silicone oil systems. We further enhance the methodology by combining accelerated thermal aging experiments with first-order kinetic modeling and Arrhenius analysis to investigate the chemical origins of gas formation. These findings fill a practical gap in diagnostic approaches for silicone oil-insulated cable terminals and contribute to future predictive maintenance practices. We hope that the revised manuscript more effectively communicates the novelty and significance of our research.

We hope this revision satisfies your concerns.

**Revision:**

**Abstract**

*With the widespread deployment of high-voltage cable terminals in power systems, insulating silicone oil has become a critical medium due to its superior dielectric and thermal properties. However, conventional diagnostic methods such as the three-ratio gas analysis developed for transformer oil have proven ineffective for silicone oil, owing to its distinct chemical structure and degradation behavior. To address this, this study aims to establish a fault-type identification method specifically for silicone oil to enhance the operational reliability of cable terminals.* *Accelerated thermal aging experiments (140 °C, 30 days) were conducted to simulate long-term aging of silicone oil. By integrating partial discharge, high-energy discharge, and breakdown experiments, the gas generation patterns of silicone oil under different stresses were systematically analyzed. Gas chromatography (GC) and infrared spectroscopy (IR) were employed to track gas composition and chemical structural changes. The results propose the following diagnostic criteria: H₂/CH₄ > 1, C₂H₄/C₂H₆ > 1, and C₂H₂/C₂H₄ < 0.1 indicate overheating faults; H₂/CH₄ < 1, C₂H₄/C₂H₆ < 0.1, and C₂H₂/C₂H₄ < 0.1 correspond to partial discharge; while H₂/CH₄ > 1, C₂H₄/C₂H₆ > 0.1, and C₂H₂/C₂H₄ > 5 signify high-energy discharge. In addition, kinetic modeling based on the Arrhenius equation was applied to extract the activation energy of pyrolytic gas formation, confirming its relation to Si–O bond cleavage. This research provides a foundation for fault diagnosis in silicone oil-insulated equipment, effectively improving the operational reliability of power systems.*

**Introduction**

*Line64-77*

*Despite the growing application of silicone oil in electrical insulation systems, effective and validated diagnostic criteria for identifying fault types in silicone oil-based equipment are still lacking. Conventional fault diagnosis methods—such as the widely used three-ratio method based on gas chromatography data (e.g., C₂H₂/C₂H₄, CH₄/H₂, and C₂H₄/C₂H₆)—have been effective for transformer oils due to their well-understood decomposition behavior under thermal and electrical stresses [8–11]. However, applying these traditional diagnostic ratios to silicone oil often produces inconsistent or misleading results, as silicone oil differs fundamentally in chemical composition, oxidation mechanisms, and thermal degradation kinetics. Notably, silicone oil generates significantly lower acetylene (C₂H₂) levels and exhibits distinct gas evolution patterns during aging and discharge processes, making transformer oil-based criteria unsuitable. These differences emphasize the urgent need to develop silicone oil-specific fault classification methods. In this context, our research proposes and experimentally validates a novel set of gas ratio indicators tailored specifically for silicone oil, aiming to address this diagnostic gap and enhance the accuracy and reliability of condition monitoring in high-voltage insulation systems.*

**Conclusions**

*This study investigates the thermal aging behavior of insulating silicone oil in high-voltage oil-filled cable terminations. Accelerated thermal aging experiments revealed that the silicone oil gradually decomposes with increasing aging time, accompanied by a continuous rise in gas content. However, Fourier-transform infrared spectroscopy (FTIR) showed no detectable bond cleavage, indicating the excellent thermal stability of the silicone oil. Based on gas chromatography analysis, we established a set of diagnostic criteria for fault classification. Specifically, when the gas content satisfies H₂/CH₄ > 1, C₂H₄/C₂H₆ > 1, and C₂H₂/C₂H₄ < 0.1, the condition is identified as overheating. A profile with H₂/CH₄ < 1, C₂H₄/C₂H₆ < 0.1, and C₂H₂/C₂H₄ < 0.1 corresponds to partial discharge, while H₂/CH₄ > 1, C₂H₄/C₂H₆ > 0.1, and C₂H₂/C₂H₄ > 5 indicate high-energy discharge. In summary, this work fills a critical gap in diagnostic techniques for silicone oil-insulated power equipment by proposing, for the first time, fault classification criteria based on GC-detectable gas ratios. These findings serve as a practical reference for the development of predictive maintenance and fault monitoring systems tailored to silicone oil applications.*

**Response to Reviewer #2:**

**General comments:**

The paper presents a timely and relevant study focusing on the diagnostic limitations of current methods for silicone oil in high-voltage cable terminals, however for improvement the following is recommended

**Answer:**

We sincerely thank the reviewer for the recognition of the relevance and timeliness of our study. We deeply appreciate the detailed and constructive feedback provided. In response, we have made substantial revisions to the manuscript to address each of your comments and enhance the scientific clarity, technical accuracy, and overall presentation of the work.

We appreciate your time and effort in reviewing the manuscript and look forward to submitting the final version with all corrections made.

We modify the manuscript with a clearer expression and show the changes made in the revised paper in red color. The specific comments were answered point by point as follows.

**Specific comments 1:**

 Although the study’s contribution is clear, the abstract could more explicitly highlight how this work advances the state of the art over existing diagnostic methods.

**Answer:**

Thank you for the suggestion. We have revised the abstract to clearly state that this study proposes a new diagnostic framework for silicone oil, which overcomes the limitations of conventional transformer oil-based diagnostic techniques.

**Revision:**

**Abstract**

*With the widespread deployment of high-voltage cable terminals in power systems, insulating silicone oil has become a critical medium due to its superior dielectric and thermal properties. However, conventional diagnostic methods such as the three-ratio gas analysis developed for transformer oil have proven ineffective for silicone oil, owing to its distinct chemical structure and degradation behavior. To address this, this study aims to establish a fault-type identification method specifically for silicone oil to enhance the operational reliability of cable terminals. Accelerated thermal aging experiments (140 °C, 30 days) were conducted to simulate long-term aging of silicone oil. By integrating partial discharge, high-energy discharge, and breakdown experiments, the gas generation patterns of silicone oil under different stresses were systematically analyzed. Gas chromatography (GC) and infrared spectroscopy (IR) were employed to track gas composition and chemical structural changes. The results propose the following diagnostic criteria: H₂/CH₄ > 1, C₂H₄/C₂H₆ > 1, and C₂H₂/C₂H₄ < 0.1 indicate overheating faults; H₂/CH₄ < 1, C₂H₄/C₂H₆ < 0.1, and C₂H₂/C₂H₄ < 0.1 correspond to partial discharge; while H₂/CH₄ > 1, C₂H₄/C₂H₆ > 0.1, and C₂H₂/C₂H₄ > 5 signify high-energy discharge. In addition, kinetic modeling based on the Arrhenius equation was applied to extract the activation energy of pyrolytic gas formation, confirming its relation to Si–O bond cleavage. This research provides a foundation for fault diagnosis in silicone oil-insulated equipment, effectively improving the operational reliability of power systems.*

**Specific comments 2:**

The nature of the discharge tests (voltage levels, duration, etc.) could be briefly summarized to enhance context.

**Answer:**

We have added a concise summary in the abstract describing the discharge test setup, including the use of a needle-plate electrode configuration and gradually increasing voltage to simulate partial discharge and breakdown conditions.

We hope this revision satisfies your concerns.

**Revision:**

**Abstract**

*With the widespread deployment of high-voltage cable terminals in power systems, insulating silicone oil has become a critical medium due to its superior dielectric and thermal properties. However, conventional diagnostic methods such as the three-ratio gas analysis developed for transformer oil have proven ineffective for silicone oil, owing to its distinct chemical structure and degradation behavior. To address this, this study aims to establish a fault-type identification method specifically for silicone oil to enhance the operational reliability of cable terminals. Accelerated thermal aging experiments (140 °C, 30 days) were conducted to simulate long-term aging of silicone oil. By integrating partial discharge, high-energy discharge, and breakdown experiments, the gas generation patterns of silicone oil under different stresses were systematically analyzed. Gas chromatography (GC) and infrared spectroscopy (IR) were employed to track gas composition and chemical structural changes. The results propose the following diagnostic criteria: H₂/CH₄ > 1, C₂H₄/C₂H₆ > 1, and C₂H₂/C₂H₄ < 0.1 indicate overheating faults; H₂/CH₄ < 1, C₂H₄/C₂H₆ < 0.1, and C₂H₂/C₂H₄ < 0.1 correspond to partial discharge; while H₂/CH₄ > 1, C₂H₄/C₂H₆ > 0.1, and C₂H₂/C₂H₄ > 5 signify high-energy discharge. In addition, kinetic modeling based on the Arrhenius equation was applied to extract the activation energy of pyrolytic gas formation, confirming its relation to Si–O bond cleavage. This research provides a foundation for fault diagnosis in silicone oil-insulated equipment, effectively improving the operational reliability of power systems.*

**Specific comments 3:**

Please avoid making general citations; instead, provide references that directly support specific claims. For example, lines 32 to 48 contain several assertions that lack citations. Be sure to support these arguments with evidence from relevant previous studies.

**Answer:**

Thank you for pointing this out. We have revised lines 32 to 53 and inserted appropriate citations from recent literature and industry standards to substantiate all claims made in the introduction.

**Revision:**

*Line40-42*

*As a result, the insulation performance of these terminals is a decisive factor in the long-term stability of cable systems [1].*

*Reference*

[1] Shaw, A.; Brown, R.; Langley, G.; Thakur, S.; Callender, G.; Andritsch, T.; Lewin, P.; Cwikowski, O. Techniques to monitor the aging of silicone oils. In: 2020 IEEE 3rd International Conference on Dielectrics (ICD); 2020; pp. 704–707.

*Line47-52*

*Moreover, silicone oil exhibits strong resistance to long-term thermal aging, effectively reducing the formation of deposits and sludge that commonly degrade insulation performance, thereby prolonging the service life of electrical equipment. Additionally, its low dielectric loss (tan δ < 0.001) and outstanding chemical stability maintain consistent electrical and mechanical protection under harsh environmental and electrical stresses [2–4].*

*Reference*

[2] Zhang, J.; Cai, Y.; Zhang, X. Deterioration characteristics of silicone oil at high voltage cable terminals in closed environment. Insul. Mat. 2023, 56 (6), 40–45.
[3] Chen, G.; Xin, X.; Song, P.; Wang, K.; Luo, L. Simulation of multi-branching streamer discharge at natural ester insulating oil-paper interface. Electr. Power Eng. Technol. 2024, 43(4): 245-252.
[4] Paul, M.; Mishra, P.; Vangapandu, D.N.; Mondal, M.; Paramane, A.; Nazir, M.T. Thermal aging characteristics of silicone rubber exposed to mineral oil. IEEE Trans. Dielectr. Electr. Insul. 2023, 30 (6), 2828–2837.

**Specific comments 4:**

Lines 51 to 54 lack citations

**Answer:**

We have added specific references to support the statements regarding the degradation behavior of silicone oil.

**Revision:**

*Line57-60*

*Experimental investigations into abnormal heating faults in 110 kV porcelain-insulated cable terminals have confirmed that increased dielectric loss (>0.005) in silicone oil results in thermal accumulation, triggering high-temperature alarms—primarily caused by inherent quality defects [5–7].*

*Reference*

[5] Zhang, R.; Xin, H.; Guo, G. Experimental study on the aging of silicone oil insulation in cable terminals of gas-insulated switchgear. High Volt. Eng. 2015, 41 (11), 3746–3752.
[6] Zhang, R.; Qiu, Z.; Wu, J.; Li, X.; Wang, S. Study on gaseous products in the aging process of silicone oil in cable terminals. In: 2018 IEEE Conference on Electrical Insulation and Dielectric Phenomena (CEIDP); 2018; pp. 394–397.
[7] Yang, X.; Zhang, J.; Song, P.; Wen, H.; Liu, Y.; Jiang, Y. Experimental study on thermal aging characters of silicone oil in cable terminal. In Proceedings of the 2020 IEEE 4th Conference on Energy Internet and Energy System Integration (EI2); IEEE: Wuhan, China, 2020; pp. 2230–2233.

**Specific comments 5:**

Avoid using numerical listings in the introduction unnecessarily. Instead, present the information in well-structured paragraphs to maintain a smooth and academic flow.

**Answer:**

The numerical list describing silicone oil properties has been rewritten as a well-structured paragraph to maintain academic flow.

**Revision:**

*Line43-52*

*To meet the increasingly stringent insulation requirements of high-voltage cable terminals, oil-filled insulation structures have become widely adopted. Among various insulating media, silicone oil has emerged as a preferred choice due to its excellent thermal stability, chemical inertness, and superior dielectric properties. Its broad operational temperature range, from −50 ℃ to 200 ℃, ensures fluidity even under extreme cold conditions. Moreover, silicone oil exhibits strong resistance to long-term thermal aging, effectively reducing the formation of deposits and sludge that commonly degrade insulation performance, thereby prolonging the service life of electrical equipment. Additionally, its low dielectric loss (tan δ < 0.001) and outstanding chemical stability maintain consistent electrical and mechanical protection under harsh environmental and electrical stresses [2–4].*

**Specific comments 6:**

When building the case for the novelty of your study, it is important to clearly identify the limitations or gaps in previous research. Support this with specific examples and explain in detail how your study addresses these shortcomings.

**Answer:**

We agree and have restructured the final paragraph of the introduction. We now explicitly state the diagnostic criteria proposed in this work and explain how they address the inadequacy of conventional transformer oil diagnostic methods when applied to silicone oil. This revision better communicates the significance and novelty of our contribution.

**Revision:**

*Line47-52*

*Moreover, silicone oil exhibits strong resistance to long-term thermal aging, effectively reducing the formation of deposits and sludge that commonly degrade insulation performance, thereby prolonging the service life of electrical equipment. Additionally, its low dielectric loss (tan δ < 0.001) and outstanding chemical stability maintain consistent electrical and mechanical protection under harsh environmental and electrical stresses [2–4].*

*Reference*

[2] Zhang, J.; Cai, Y.; Zhang, X. Deterioration characteristics of silicone oil at high voltage cable terminals in closed environment. Insul. Mat. 2023, 56 (6), 40–45.
[3] Chen, G.; Xin, X.; Song, P.; Wang, K.; Luo, L. Simulation of multi-branching streamer discharge at natural ester insulating oil-paper interface. Electr. Power Eng. Technol. 2024, 43(4): 245-252.
[4] Paul, M.; Mishra, P.; Vangapandu, D.N.; Mondal, M.; Paramane, A.; Nazir, M.T. Thermal aging characteristics of silicone rubber exposed to mineral oil. IEEE Trans. Dielectr. Electr. Insul. 2023, 30 (6), 2828–2837.

*Line57-60*

*Experimental investigations into abnormal heating faults in 110 kV porcelain-insulated cable terminals have confirmed that increased dielectric loss (>0.005) in silicone oil results in thermal accumulation, triggering high-temperature alarms—primarily caused by inherent quality defects [5–7].*

*Reference*

[5] Zhang, R.; Xin, H.; Guo, G. Experimental study on the aging of silicone oil insulation in cable terminals of gas-insulated switchgear. High Volt. Eng. 2015, 41 (11), 3746–3752.
[6] Zhang, R.; Qiu, Z.; Wu, J.; Li, X.; Wang, S. Study on gaseous products in the aging process of silicone oil in cable terminals. In: 2018 IEEE Conference on Electrical Insulation and Dielectric Phenomena (CEIDP); 2018; pp. 394–397.
[7] Yang, X.; Zhang, J.; Song, P.; Wen, H.; Liu, Y.; Jiang, Y. Experimental study on thermal aging characters of silicone oil in cable terminal. In Proceedings of the 2020 IEEE 4th Conference on Energy Internet and Energy System Integration (EI2); IEEE: Wuhan, China, 2020; pp. 2230–2233.

**Specific comments 7:**

In subsections 2.1 and 2.2, there are several instances of incorrect word breaks or hyphenation errors that affect readability and clarity. For example, 'ox-ygen atoms' should be corrected to 'oxygen atoms', 'ac-celerated' to 'accelerated', 'pro-cess' to 'process', and 'sin-gle-measurement' to 'single measurement'. Please carefully proofread all of subsections 2.1 and 2.2 to eliminate such typographical issues and ensure smooth and professional language throughout.

**Answer:**

Thank you for pointing this out. We have carefully reviewed and corrected all incorrect word breaks and hyphenation issues in Sections 2.1 and 2.2. Specific terms such as “ox-ygen,” “ac-celerated,” “pro-cess,” and “sin-gle-measurement” have been revised to “oxygen,” “accelerated,” “process,” and “single measurement,” respectively. The hyphenation issues in the text have been fully addressed, while the word breaks at line endings cannot be manually adjusted due to typesetting constraints. Additional proofreading was conducted to ensure consistent and professional formatting throughout these sections.

We hope this revision satisfies your concerns.

**Revision:**

*Line80-85*

*The silicone oil sample selected for testing was AK-50 insulating silicone oil produced by Wacker Chemie AG, Germany. Its primary component is polydimethylsiloxane (PDMS), featuring a linear chain structure composed of alternating silicon and oxygen atoms, with each silicon atom bonded to two methyl groups. The basic molecular structure is illustrated in Fig 1. The key physical and chemical properties of the silicone oil sample are summarized in Table 1(The technical datasheet provided by the manufacturer, Wacker Chemie AG).*

**Specific comments 8:**

In lines 90 to 91, you mention: 'During the long-term high-temperature aging process of 30 days, samples were taken every 6 hours, 1 day, 5 days, and subsequently every five days.' However, the rationale for selecting these specific sampling intervals is not explained. Please clarify the basis for choosing these time points whether they are based on previous studies, preliminary tests, or specific degradation behavior expected in silicone oil. Providing justification will help readers understand the experimental design and its relevance to the study’s objectives.

**Answer:**

We concur with the reviewer's perspective that providing theoretical rationales strengthens experimental design. Based on preliminary studies and the known degradation behavior of silicone oil, we have clarified the reasoning behind selecting sampling intervals at 6 hours, 1 day, 5 days, and subsequently every 5 days. Short-term intervals (6 hours, 1 day) aim to capture rapid changes during the early-stage thermal decomposition. Extended intervals (≥5 days) monitor steady-state gas accumulation and long-term aging processes under sustained thermal exposure.

We hope this revision addresses your concerns comprehensively.

**Revision:**

*Line100-103*

*During the long-term high-temperature aging process of 30 days, samples were taken every 6 hours, 1 day, 5 days, and subsequently every five days (Short-term intervals (6 h, 1 d) aim to capture rapid changes in the early stages of thermal decomposition, while the longer intervals (≥5 d) are used to monitor steady-state gas accumulation and long-term aging progression).*

**Specific comments 9:**

After reviewing the Results and Discussion sections, it is evident that your arguments are not sufficiently supported by references to previous studies. While the Introduction does mention some weaknesses in existing research, this context is not carried through into the discussion of your findings. This weakens the overall strength of your claims. I strongly recommend revisiting the Results and Discussion sections to compare your findings with those of previous studies. Supporting your interpretations with established literature will help validate your conclusions and clearly demonstrate the contribution and novelty of your work.

**Answer:**

Thank you for this insightful suggestion. We have revised the Results and Discussion sections to incorporate comparisons with previous studies.

**Revision:**

*Line306-319*

*Based on the experimental data, the following conclusions can be drawn: For silicone oil, if the gas content satisfies the condition H₂/CH₄ > 1, C₂H₄/C₂H₆ > 1, and C₂H₂/C₂H₄ < 0.1, it can be considered as an overheating fault. If the gas content satisfies H₂/CH₄ < 1, C₂H₄/C₂H₆ < 0.1, and C₂H₂/C₂H₄ < 0.1, it can be identified as partial discharge. Additionally, if the gas content satisfies H₂/CH₄ > 1, C₂H₄/C₂H₆ > 0.1, and C₂H₂/C₂H₄ > 5, it indicates high-energy discharge. These diagnostic criteria represent a significant advancement over traditional transformer oil fault diagnosis methods, which commonly use the three-ratio method (C₂H₂/C₂H₄, CH₄/H₂, and C₂H₄/C₂H₆) to identify fault types. However, these ratios, while effective for transformer oil, do not yield reliable results when applied to silicone oil due to its distinct chemical structure and decomposition behavior. For silicone oil, the relative production of gases such as acetylene (C₂H₂) is much lower, and the gas evolution patterns are fundamentally different. This study provides a set of gas-ratio indicators specifically tailored for silicone oil, filling the gap in existing fault diagnosis methodologies and offering a foundation for more accurate fault identification in silicone oil-insulated equipment.*

**Specific comments 10:**

When summarizing detailed results such as those from FTIR analysis, consider presenting the key findings in a table for improved clarity and readability. A well-organized table can help highlight the main spectral changes, functional group variations, and trends across conditions more effectively than text alone, making it easier for readers to grasp and compare the results.

**Answer:**

Thank you for this constructive suggestion. We have summarized the FTIR results in a new table (Table X), which presents each major absorption peak along with its vibrational assignment, associated functional groups, and interpretation. This tabular format improves clarity and allows readers to easily identify key chemical changes in the silicone oil structure under aging and discharge conditions.

We hope this revision satisfies your concerns.

**Revision:**

***Table 6. Summary of Main FTIR Absorption Peaks and Functional Group.***

| *Wavenumber (cm⁻¹)* | *Vibrational Mode* | *Functional Group / Source* | *Interpretation / Structural Implication* |
| --- | --- | --- | --- |
| *2962* | *C–H stretching* | *CH₃, CH₂ (alkanes)* | *Indicates saturated hydrocarbon content* |
| *1412* | *C–H bending* | *CH₃, CH₂ (alkanes)* | *Typical of methyl/methylene groups* |
| *1257* | *C–O–C stretching* | *Ester, ether, C–O in polymers* | *Suggests presence of oxygen-containing polymer structures* |
| *1079* | *Si–O–Si stretching* | *Siloxanes, SiO₂* | *Characteristic peak of silicone backbone* |
| *1008* | *C–O stretching* | *Ether or alcohol groups* | *Indicates oxidative structural modification* |
| *864* | *Si–OH bending* | *Silanol (Si–OH)* | *Reflects hydrolysis or aging-induced silanol formation* |
| *785* | *Si–C stretching* | *Organosilicon compounds* | *Characteristic of organic–inorganic hybrid linkages* |
| *685，660* | *Si–OH bending (double peak)* | *Siloxanes, silicone rubber* | *Common in aged or moisture-exposed silicone structures* |
| *502* | *Si–O bending (deformation)* | *Si–O network* | *Low-frequency bending of siloxane framework* |

**Specific comments 11:**

Equations are not well presented example equations 1 to 3.

**Answer:**

Thank you for pointing this out. We have revised Equations (1) to (3) using standard mathematical formatting. Each equation is now centered, properly numbered.

**Revision:**

$$\frac{dC}{dt}=k\times\left( C_{max}-C \right) (1)$$

$$\ln\left( \frac{C_{max}}{C_{max}-C} \right)=kt (2)$$

$$k=A\times e^{-\frac{E_{a}}{RT}} (3)$$

**Specific comments 12:**

Please refrain from using numerals to summarize your work; instead, present your findings in a well-organized paragraph. Your conclusion can also reflect your thoughts on the broader field and highlight the contributions your study makes to this area of expertise.

**Answer:**

Thank you for this important suggestion. We have revised the conclusion by removing the enumerated list format and rewriting the section as a cohesive, well-structured paragraph. This change improves the academic tone and readability of the manuscript.

We hope this revision satisfies your concerns.

**Revision:**

Conclusion

*This study investigates the thermal aging behavior of insulating silicone oil in high-voltage oil-filled cable terminations. Accelerated thermal aging experiments revealed that the silicone oil gradually decomposes with increasing aging time, accompanied by a continuous rise in gas content. However, Fourier-transform infrared spectroscopy (FTIR) showed no detectable bond cleavage, indicating the excellent thermal stability of the silicone oil. Based on gas chromatography analysis, we established a set of diagnostic criteria for fault classification.* *Specifically, when the gas content satisfies H₂/CH₄ > 1, C₂H₄/C₂H₆ > 1, and C₂H₂/C₂H₄ < 0.1, the condition is identified as overheating. A profile with H₂/CH₄ < 1, C₂H₄/C₂H₆ < 0.1, and C₂H₂/C₂H₄ < 0.1 corresponds to partial discharge, while H₂/CH₄ > 1, C₂H₄/C₂H₆ > 0.1, and C₂H₂/C₂H₄ > 5 indicate high-energy discharge.* *In summary, this work fills a critical gap in diagnostic techniques for silicone oil-insulated power equipment by proposing, for the first time, fault classification criteria based on GC-detectable gas ratios. These findings serve as a practical reference for the development of predictive maintenance and fault monitoring systems tailored to silicone oil applications.*
