# Supplementary material for: Research on aging behaviors of insulating silicone oil for cable terminals based on chromatographic and spectroscopic analysis
Source: PLoS One. 2025 Nov 7;20(11):e0334552. doi: 10.1371/journal.pone.0334552 (PMC12594329; doi:10.1371/journal.pone.0334552)
Supplement: Attachment — Submitted filename: Response_to_Reviewers_auresp_2.docx [file pone.0334552.s005.docx]

**Revision Report for Manuscript** **PONE-D-25-13265R1**

**Dear Editors:**

We gratefully appreciate both editors and reviewers for their careful reading, helpful comments, and constructive suggestions, which have significantly improved the quality of our manuscript entitled “**Research on Aging and Oil Chromatographic Characteristics of Insulating Silicone Oil for Cable Terminals**” (**Manuscript Number: PONE-D-25-13265R1**).

We have carefully considered all comments from the reviewers and revised our manuscript accordingly. In the following section, we summarize our responses to each comment from the reviewers. We believe that our responses have well addressed all concerns from the reviewers. Here, we would like to re-submit the revised manuscript to *PLOS ONE*. Hope these revisions could present a better view to the readers and make the paper acceptable for publication in the journal.

Looking forward to hearing from you soon. Have a nice day.

Best regards,

The Authors

Electric Power Research Institute of State Grid Jiangsu Electric Power Co,

Nanjing, 211103, China

+86-17805166516

zhangweijsdl@163.com

**Response to Reviewer #1:**

**General comments:**

Some improvements have been made; however, there are still some areas which needs further improvements.

**Answer:**

Thank you very much for your positive feedback and kind words regarding our revisions. We are grateful for your continued support and thoughtful comments, which have significantly contributed to strengthening the manuscript. We will carefully address all the comments and make the necessary adjustments to ensure the paper meets the required standards. We appreciate your time and effort in reviewing the manuscript and look forward to submitting the final version with all corrections made.

We have modified the manuscript with a clearer expression and show the changes made in the revised paper in red color. The specific comments were answered point by point as follows.

**Specific comments 1:**

The author should define the colours used in Figure 1, for example red colour represent Oxygen atom etc. Use the standard colour for each atom.

**Answer:**

Thank you for the valuable suggestion. The colours used in Figure 1 have now been defined in the figure caption. We have also ensured that standard colour conventions are followed(e.g., red for oxygen, white for hydrogen, grey for carbon, etc.).

**Revision:**

Fig 1. Chemical structure of polydimethylsiloxane as the primary component in AK-50 insulating silicone oil

**Specific comments 2:**

I recommend Figure 1 caption to read “Chemical structure of polydimethylsiloxane as the primary component in AK-50 insulating silicone oil”.

**Answer:**

Thank you for the helpful suggestion. We have revised the caption of Figure 1 to read: “Chemical structure of polydimethylsiloxane as the primary component in AK-50 insulating silicone oil’’ as recommended.

**Revision:**

Fig 1. Chemical structure of polydimethylsiloxane as the primary component in AK-50 insulating silicone oil

**Specific comments 3:**

There are still some typo errors for example, no space between a number and a unit in 250℃ and 55℃ (Table 1). Make the necessary corrections throughout your manuscript.

**Answer:**

Thank you for pointing this out. We have carefully checked the manuscript and corrected all instances of missing spaces between numbers and units (e.g., “250℃” changed to “250 ℃”) in Table 1 and throughout the manuscript as necessary.

**Revision:**

Table 1. Basic parameters of test sample silicone oil.

| **Basic parameters** | **Performance index** |
| --- | --- |
| Density (25 ℃) | 0.96 g/cm^3^ |
| Kinematic Viscosity | 50 mm^2^ s^-1^ |
| Coefficient of thermal expansion at 0-150 ℃ | 9.5 cm^3^/ (cm^3^ ℃ )×10^-4^ |
| Thermal conductivity at 50 ℃ | 0.15 W K^-1^ m^-1^ |
| Flash point | >250 ℃ |
| Pour point | 55 ℃ |
| Volatility | <2% |

**Specific comments 4:**

I recommend Figure 2 caption to read “Pictorial representation of (a) oven and (b) silicone oil at different aging stages”.

**Answer:**

Thank you for the helpful suggestion. We have revised the caption of Figure 2 to read: “Pictorial representation of (a) oven and (b) silicone oil at different aging stages”as recommended.

**Revision:**

(a) oven	(b) silicone oil at different aging stages

Fig 2. Pictorial representation of (a) oven and (b) silicone oil at different aging stages

**Specific comments 5:**

I recommend Figure 3 caption to read “Schematic diagram of (a) partial discharge experimental circuit and (b) physical drawing of needle plate electrode”.

**Answer:**

Thank you for the insightful suggestion. We have revised the caption of Figure 3 to read: “Schematic diagram of (a) partial discharge experimental circuit and (b) physical drawing of needle plate electrode”as recommended.

**Revision:**

(a) partial discharge experimental circuit (b) physical drawing of needle plate electrode

Fig 3. Schematic diagram of (a) partial discharge experimental circuit and (b) physical drawing of needle plate electrode

**Specific comments 6:**

I recommend Figure 3 caption to read “Oil chromatography data for continuous aging over 30 days for (a) CO₂, CO, and H₂; and (b) C₂H₆, C₂H₄, and CH₄”.

**Answer:**

Thank you for the helpful suggestion. We have revised the caption of Figure 3 for clarity and consistency with the manuscript. It now reads: “Oil chromatography results over 30 days of aging: (a) CO₂, CO, and H₂; (b) C₂H₆, C₂H₄, and CH₄”.

**Revision:**

(a) CO₂, CO, and H₂

(b) C₂H₆, C₂H₄, and CH₄.

Fig 5. Oil chromatography data for continuous aging over 30 days for (a) CO₂, CO, and H₂; and (b) C₂H₆, C₂H₄, and CH₄

**Specific comments 7:**

Use the correct unit for wavenumber (cm⁻¹) in Figures 6 and 7, apply superscript and use the correct minus sign from symbols.

**Answer:**

Thank you for pointing this out. We have corrected the unit for wavenumber in Figures 6 and 7 to “cm⁻¹” using the appropriate superscript format and the correct minus sign symbol. These changes have been applied consistently throughout the figures and captions.

**Revision:**

Fig 6. Infrared Spectrum of AK50 Silicone Oil and Aging Data Over 30 Days.

**Specific comments 8:**

Figures 6 and 7 can be drawn in the same graph; how can the values of Transmittance (Figure 7) be negative?

**Answer:**

Thank you for your valuable comment. In the revised manuscript, Figures 6 and 7 have been combined into a single graph for better comparison, as suggested. Regarding the negative transmittance values in the original Figure 7, we would like to clarify that transmittance itself cannot be negative; the apparent negative values were due to a vertical offset applied during plotting for visual clarity. This issue has been corrected in the revised figure to avoid any confusion.

**Revision:**

Fig 6. Infrared Spectrum of AK50 Silicone Oil and Aging Data Over 30 Days.
